# Non-technical skills for urological surgeons (NoTSUS): development and evaluation of curriculum and assessment scale

**DOI:** 10.1007/s00345-020-03406-6

**Published:** 2020-08-18

**Authors:** Abdullatif Aydın, Cora M. Griffin, Oliver Brunckhorst, Ahmed Al-Jabir, Nicholas Raison, Haleema Aya, Craig McIlhenny, James Brewin, Majid Shabbir, Joan Palou Redorta, Muhammad Shamim Khan, Prokar Dasgupta, Kamran Ahmed

**Affiliations:** 1 MRC Centre for Transplantation, Guy’s Hospital, King’s College London, 5th Floor Southwark Wing, London, SE1 9RT UK; 2 Department of Urology, NHS Forth Valley, Glasgow, UK; 3 Department of Urology, Salisbury NHS Foundation Trust, Salisbury, UK; 4 Department of Urology, Guy’s and St, Thomas’ NHS Foundation Trust, London, UK; 5 European School of Urology, European Association of Urology, Amsterdam, The Netherlands; 6 Dept. of Urology, Fundació Puigvert, Universitat Autònoma de Barcelona, Barcelona, Spain; 7 Department of Urology, King’s College Hospital NHS Foundation Trust, London, UK

**Keywords:** Non-technical skills, Simulation, Urology, NOTSS, NoTSUS

## Abstract

**Objective:**

In the last decade non-technical skills (NTS) have emerged as a vital area for improvement within surgery. This study aims to develop and evaluate a Non-technical Skills for Urological Surgeons (NoTSUS) training curriculum and assessment scale.

**Methods:**

This international, longitudinal and observational study began with a 3-round Delphi methodology to refine curriculum contents and rating scale. Sessions with up to four participants were delivered where each candidate undertook an independent scenario within the validated full immersion simulation environment. Candidates were assessed using both the NoTSS (Non-technical Skills for Surgeons) and NoTSUS rating scales by NTS-trained and non-trained experts. A post-training evaluation survey was distributed.

**Results:**

62 participants comprising trainees (*n* = 43) and specialists (*n* = 19) undertook the NoTSUS course. The NoTSS and NoTSUS scales correlated well, with a mean difference of 3.3 in the overall total (*p *= 0.10, r = 0.53). However, there was significant differences in scores between the NoTSS-trained and non-trained raters (*n* = 28, *p* = 0.03). A one-way ANOVA test revealed significant improvement throughout the four simulation scenarios in each session (*p* = 0.02). The NoTSUS curriculum received positive feedback from participants and demonstrated educational value and acceptability.

**Conclusions:**

The NoTSUS curriculum has demonstrated high educational value for NTS training aimed at urologists, with marked improvement throughout sessions. Correlation of NoTSUS and NoTSS scales proves its suitability for evaluating NTS in future training. Demonstration of inter-rater reliability indicates that the scale is reliable for use in assessment by expert faculty members. Furthermore, qualitative feedback from participants suggests gain of transferrable skills over the course.

**Electronic supplementary material:**

The online version of this article (10.1007/s00345-020-03406-6) contains supplementary material, which is available to authorized users.

## Introduction

Non-technical skills (NTS) are the cognitive and social abilities that complement a clinician’s technical ability, comprising decision-making, leadership, team work, and situational awareness [1, 2]. They are often grouped into social skills, cognitive skills, and personal resource factors. Social skills refer to leadership, communication and teamwork, with communication being a particularly important factor as it is one of the biggest causes for surgical errors. Leadership itself can be subdivided into task and resource management, decision-making, and maintaining standards. Within cognitive skills lie decision-making, planning and situational awareness. Decision-making is often thought of as a more complex skill, which is accumulated throughout training as a surgeon gains experience and knowledge on which to base their decisions. Personal resource factors include the capacity of an individual to cope with stressors and fatigue, which have been shown to negatively impact technical skills in the operating room (OR).

There are many stressors in surgery, such as distractions and complications, so a surgeon must be trained to minimise their effect on performance [2]. Factors such as lack of sleep, causing fatigue, can result in a higher number of clinical errors and weaken leadership skills [3]. In fact, all NTS components interlink and affect each other, and they may be trained either individually or concurrently to result in improved performance in the OR [2].

A standardised and validated NTS training program is currently lacking in the literature for urologists to achieve better training [1, 4, 5]. With the hypothesis that a urology-focused NTS training course would be well received and provide transferrable skills, the aims of this study are: (1) to develop a simulation-based NTS curriculum for the training and assessment of urological surgeons and (2) assess the validity and reliability evidence, as well as the educational impact, of the developed curriculum. Furthermore, we aim to evaluate the validity evidence of the developed assessment scale.

## Methods

Ethical approval was obtained, as part of the Simulation in Urological Training and Education (SIMULATE) project (BDM/14/15-68) [6].

### Curriculum development

This prospective, international, longitudinal and observational study selected ureteroscopy (URS) as an index procedure for the technical component of the curriculum given the availability of training models [7]. Initially, to refine the curriculum content and scenarios, a two-round Delphi was conducted whereby questionnaires were sent round to trainees (i.e. urology residents-in-training) and specialists (i.e. board-qualified urologists), including experts in NTS and urolithiasis procedures. The first round involved a total of 47 respondents, 23 of whom were specialists. The second round involved 8 specialists. The Non-technical Skills for Urological Surgeons (NoTSUS) assessment scale, a modified version of the Non-technical Skills for Surgeons (NoTSS) scale, was also developed using data from the first two rounds, and then involved a final, third round with two NoTSS-trained urology experts (A and B) to finalise the content (Supplementary Appendix).

### Study process and simulation

**Fig. 1:**
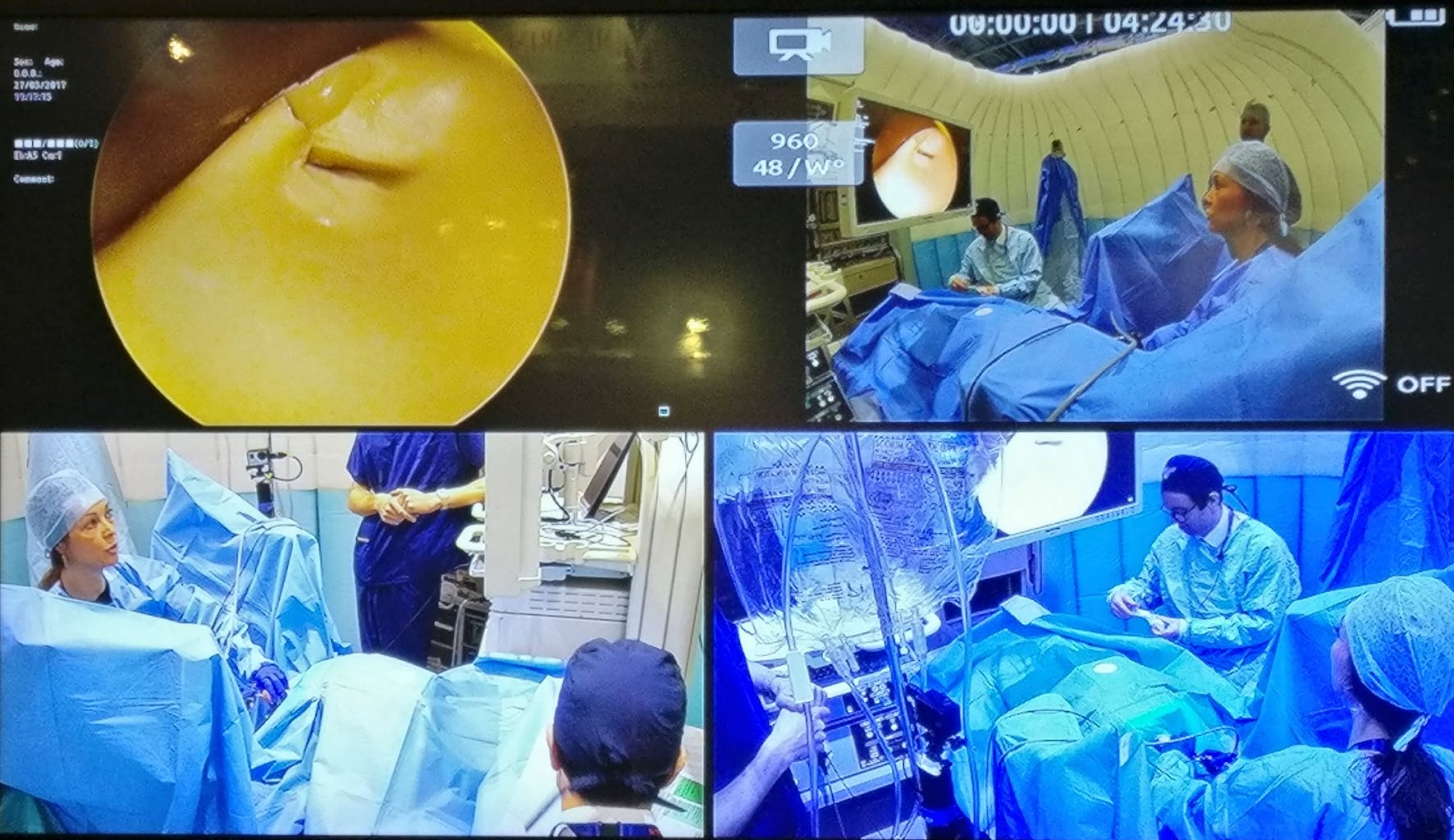
A view from training sessions within the Full Immersion Simulation ‘Igloo’ environment

The developed curriculum was delivered as hands-on training courses in the UK and to international attendees at the European Association of Urology (EAU) Annual Congress. A total of 17 2-h training sessions with 3–4 participants were delivered on four independent occasions in Manchester (*n* = 14, 4 sessions), London (*n* = 4, 1 session), during London EAU 2017 (*n* = 24, 6 sessions) and Copenhagen EAU 2018 (*n* = 20, 6 sessions). Each candidate took turns to undertake an independent scenario within the previously validated full immersion simulation (FIS) ‘Igloo’ environment (Fig. 1; Imperial College, London, UK) [8]. Participants were required to fully gown and glove during simulation sessions. Other team members including anaesthetist, assistant nurse and floating nurse were role-played, to improve fidelity. Cameras were integrated into the setup in order to record candidates for later video assessment. The previously validated Uro-Scopic Trainer model (Limbs and Things, UK) [9] was utilised for URS technical performance during scenarios. Two faculty members, with expertise in urolithiasis and NTS training, supervised participants. Each participant undertook a 15–20 min scenario whilst faculty and the remaining participants in each group observed sessions through a video-link. Debriefing followed scenarios in a structured manner, utilising the NoTSUS parameters and providing focused feedback.

### Performance evaluation

Each candidate was assessed using both the NOTSS and NoTSUS rating scales. Seven sessions were led by the NTS-trained experts A and B who performed assessments in real-time and the remaining ten sessions were led by other senior urologists (*n* = 4), utilising the same feedback format. Where NTS-trained experts were absent for supervision, detailed videos of these sessions (*n* = 10) were provided to them for assessment (as seen in Fig. 1). Expert A assessed participants from all sessions (*n* = 57) and Expert B assessed participants from 11 sessions (*n* = 40). The non-trained experts provided assessment scores for 29 participants. An evaluation questionnaire was distributed to participants after the simulation to evaluate the NoTSUS course and self-perceived improvement, employing a 5-point Likert scale for quantitative feedback and comment boxes for qualitative feedback.

### Outcome measures

Primary outcome measures were validity, reliability and educational value. Validity was measured by improvement in candidate performance over the course judged by NoTSUS score. Reliability parameters included the measurement of inter-rater reliability and the comparison of the NoTSUS scale with the gold-standard NoTSS scale. The perceived educational value was judged using candidate responses to the post-simulation questionnaire. Secondary measures included the fidelity of the scenarios, again judged by participants’ responses on the questionnaire.

### Statistical analysis

Microsoft Excel (Redmond, WA, USA) was used to collate all quantitative and qualitative data. Descriptive statistics were used for questionnaire data. Pearson’s correlation coefficient and paired t-tests were utilised to investigate correlation and agreement, respectively, in scores for inter-rater reliability, using SPSS^®^ Statistics version 26 (IBM^®^, Armonk, NY, USA). The former was also used for correlation between NoTSUS vs NoTSS scales. GraphPad Prism version 8 (San Diego, CA, USA) was utilised to demonstrate all graphs and perform other basic statistical analyses. Unpaired t-tests were performed for the differences between populations. A one-way ANOVA test was used to measure improvement in NoTSUS scores over the four simulation scenarios in each session. Tukey’s multiple comparisons test was then used to further investigate the improvement of the cohort comparing sessions 1 and 3, and sessions 1 and 4. A *P* value < 0.05 was considered to be statistically significant for all tests.

## Results

The Delphi study resulted in the development of four scenarios: (1) briefing the emergency OR team and performing an adequate WHO checklist; (2) management of an intra-operative septic shock emergency; (3) interacting with an inexperienced scrub nurse and (4) troubleshooting with faulty instrumentation. The NoTSUS assessment scale was adapted into a 5-point Likert scale (as opposed to the 4-point NoTSS scale) by experts for ease of marking and further expanded into five sections (Supplementary Appendix).

### Demographics

Overall, 62 participants received NoTSUS training consisting of trainees (*n* = 43) and specialist (*n* = 19) surgeons with a wide range of experience (1–7 years of training and 1–20 years of specialist practice) from all over the globe. The mean age of participants was 33.7 years (range: 24–57 years) with 27% female participation. Neither level of ureteroscopy experience (*p* = 0.28) nor level of overall clinical experience (*p* value = 0.78), determined by training years, was found to significantly impact mean NoTSUS scores. There were no statistically significant differences found in the scores between participants who had previously undertaken a form of NTS training (*n* = 15) and those who had not (*n* = 47; *p* = 0.57).

### Correlation of scales and assessors

**Fig. 2:**
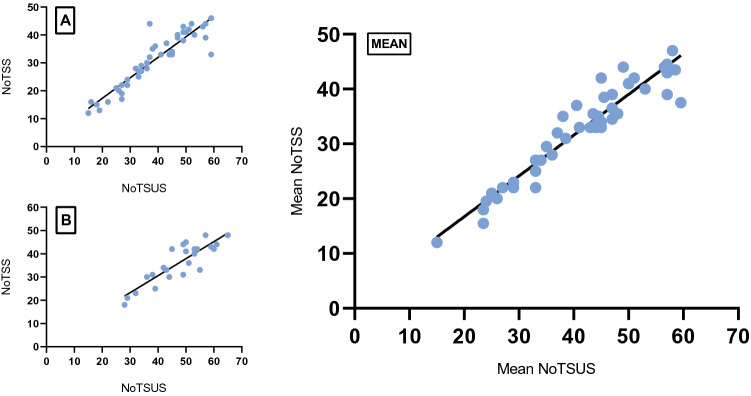
Correlation between the Non-technical Skills for Surgeons (NoTSS) and Non-technical Skills for Urological Surgeons (NoTSUS) scales amongst both raters A and B

**Fig. 3:**
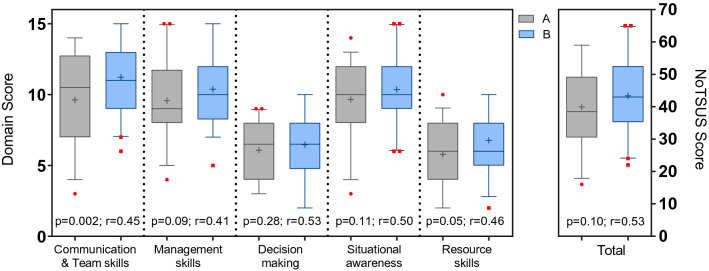
Inter-rater reliability of all NoTSUS domains and total between the two expert raters

Correlation of the NoTSS and NoTSUS scores for each participant gave a correlation coefficient of *r* = 0.93 and *r* = 0.88, respectively, for expert raters A (*n* = 43, *p* < 0.0001) and B (*n* = 26, *p* < 0.0001) (Fig. 2). The two expert raters did not significantly differ in their scoring (Fig. 3), with a mean difference of 3.3 points in the overall total, as confirmed by a paired* t*-test of candidate scores (*n* = 36, *p* = 0.10, *r* = 0.53). Scores showed moderate correlation throughout all domains of NoTSUS. However, there was a significant difference in mean scores (*n* = 28, *p* = 0.03) between the NoTSS-trained (*n* = 2) and non-trained raters (*n* = 4).

### Improvement between sessions

**Fig. 4:**
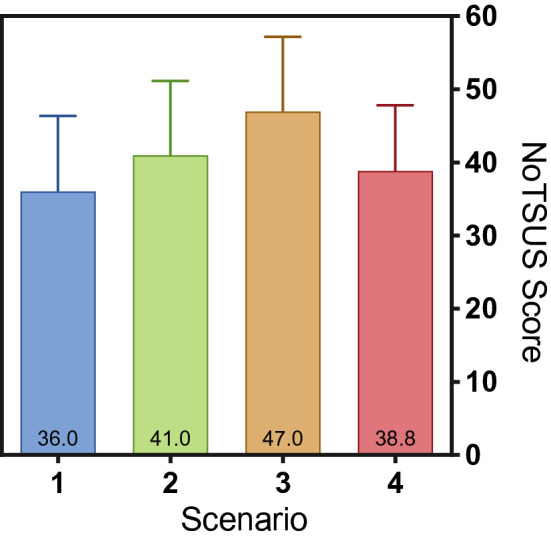
Improvement in mean NoTSUS scores over consecutive scenarios (*p* = 0.02)

A one-way ANOVA test revealed significant improvement throughout the four simulation scenarios in each session (*p* = 0.04; Fig. 4). Participants demonstrated marked improvements between sessions 1 and 3, with a mean improvement of 10.9 points (*p* = 0.03), but there was no statistically significant improvement noted between other sessions.

### Educational value

**Fig. 5:**
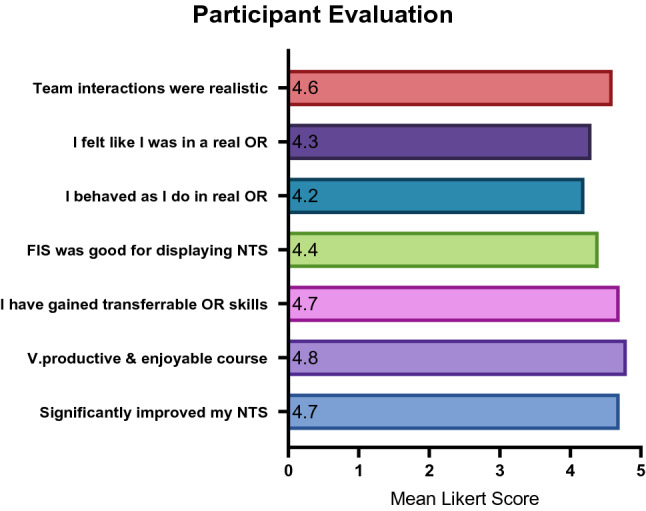
Post-training evaluation survey of educational value (Likert scale 1–5)

The course was well received by participants (Fig. 5). The mean score on a 5-point Likert scale for all parameters asked about in the post-simulation survey was 4.5 out of 5. Individual aspects of FIS received an average mean Likert score of 4.4 for realism, including for lighting (4.24/5), sound (4.34/5), posters (4.10/5), anaesthetist (4.66/5), scrub nurse (4.56/5) and scenario (4.65/5). Qualitative feedback received about FIS included that it ‘was a highlight’ of training, had ‘well-led debriefing sessions’, a ‘good, varied range of scenarios’ and ‘great discussion points’. There was also enthusiasm with regard to propagating FIS and setting it up in further areas. Generally, feedback reflected that the course had ‘brilliant learning points’ and ‘should be mandatory for all’. One participant reported that they ‘will pay far more attention to [NTS] in practice’ and it helped with ‘awareness’ of NTS. Video feedback was appreciated and was a ‘very well organised, productive day’.

## Discussion

NTS training has developed considerably in the past decade, with many more courses and validated training tools becoming available. Surgical simulation literature has mainly focused on trainees and medical students rather than experienced surgeons. However, experienced surgeons and those post-fellowship may also become exhausted and experience burn-out due to poor NTS training [10, 11]. As NTS do not always correlate with experience, there is evidently room for training to be incorporated at higher stages of medical training [10–12]. This study aimed to add to the current evidence-base with the NoTSUS training course.

In this study, the level of experience of participants did not significantly affect their NTS performance, whether it was measured by number of URS procedures or overall clinical experience (in years). Although this does not demonstrate construct validity, it supports the hypothesis that despite having superior technical skills as experienced surgeons, there is scope for improvement of NTS at all levels of training [10, 11]. However, some studies do show a difference in NTS between junior and senior participants [13–15]. This contradiction is likely due to the wide variation between studies, which look at very different sample sizes and populations, and use different scenarios and examiners to test candidates.

In contrast to previous studies, 36% of participants had previously received some form of NTS training and 19% had also been assessed, either formally or informally. This shows a positive trend in the availability and popularity of training [16]. However, no difference was shown between participants who had previously undertaken a NTS training course and those who had not. Although the style of NTS training undertaken previously varied greatly, one can speculate that courses are either not specialised enough to improve NTS, or that participants do not retain the skills they develop on courses. Despite some studies claiming that participants retain skills for up to 6 months, others note no difference if previous NTS training has been undertaken [11, 16, 17].

The NoTSUS rating scale is significantly correlated to the extensively validated NoTSS scale, proving its suitability for evaluating NTS. This scale is therefore appropriate for continued use in further NTS training curricula. Inter-rater reliability is demonstrated by comparison of the NoTSUS scores of the two independent expert examiners. A more detailed investigation should be attempted between a wider pool of examiners to confirm inter-rater reliability. However, there was significant differences between faculty members who had not received NoTSS training and the two NTS experts, highlighting the value of training the trainers.

The overall improvement in the scores of participants with consecutive scenarios in each group of four highlights the value of effective debriefing between scenarios. Based on their NoTSUS scores, participants did not make a significant improvement between scenarios one and four (the first and last scenarios undertaken), perhaps due to less numbers undertaking scenario four (*n* = 11) as opposed to the former three scenarios (*n* = 17). This may also be due to other factors such as fatigue or loss of interest. Debriefing aspect of the course was poorly documented and there is no feedback on the process. This could be improved for the NoTSUS curriculum by using a validated framework [18], such as the ‘Diamond’ framework [18–23], which is specifically designed to help assessors focus on NTS; this may be important if less experienced faculty are employed for teaching. Candidates observing one another during each of their scenarios is clearly very helpful; participants in other studies agree that it is a good learning opportunity [24]. OR-based debriefing would be a useful transferable skill from this training to potentially improve retention of skills [25]. Resources should be created or modified for this purpose, as it differs from simulation debriefing and must fit other logistical criteria such as being brief to fit in with the busy OR schedule [26].

The overwhelmingly positive feedback given about the NoTSUS course and the use of FIS is a reason to continue to improve and expand the curriculum in future, and to keep improving the number of trainees with exposure to NTS training. The self-perceived improvement of candidates, together with the positive feedback and overall satisfaction with the realism of the scenarios, demonstrates educational value and acceptability of the course.

### Limitations

This study also has a number of limitations. Firstly, recruited participants were heterogenous in terms of experience and exposure to NTS and in different phases of their careers. In its current form, the NoTSUS course is aimed at all levels of urologists. Although less time consuming, training multiple different levels of expertise at once in this way may reduce teaching quality for seniors; future training could benefit from further specialised scenarios for higher and lower levels [27]. Furthermore, there were six sessions which only had 3 participants, which may have played a role in the limited results attained in improvement through sessions. Secondly, training sessions were not led by the same faculty members. This may have affected delivery of the curriculum. The NTS experts were not able to assess all sessions and utilise both assessment scales for all. This may have affected the results of this study. Finally, participants should subsequently be assessed in the OR, to evaluate transfer of skills.

## Conclusion

In summary, the developed NoTSUS curriculum is a useful addition to surgical training, and its analysis adds relevant information to the ever-growing literature on NTS training in healthcare. The NoTSUS scale was able to reliably mark participants for scenarios, correlating to the validated NoTSS scale and demonstrating inter-rater reliability. Improvement of skills over the course was demonstrated through total NoTSUS scores and all participants identified self-perceived improvement and satisfaction in all areas of the course.

## Electronic supplementary material

Below is the link to the electronic supplementary material.

Supplementary material 1 (PDF 557 kb)
