# Supplementary material for: Non-technical skills for urological surgeons (NoTSUS): development and evaluation of curriculum and assessment scale
Source: World J Urol. 2020 Aug 18;39(6):2231–7. doi: 10.1007/s00345-020-03406-6 (PMC8217036; doi:10.1007/s00345-020-03406-6)
Supplement: Supplementary file 1 — Supplementary material 1 (PDF 557 kb) [file 345_2020_3406_MOESM1_ESM.pdf]

SIMULATE
Simulation in Urological Training & Education

KING'S College LONDON

Non-technical Skills for Urological Surgeons (NoTSUS)

Candidate Name: …………………………. **Date:** …./…./ 20….

Assessor's Name: ………………………….

Process	Non-Technical Skills Assessment Tool	NA	1	2	3	4	5
Communication and Team Skills	Appropriate verbal communication with anaesthetist, surgical nurse and radiographer						
	Gives adequate information to respective team members						
	Receptive to information received						
Management Skills	Leads and manages the team appropriately						
	Good use of resources (e.g. radiographer) and management of workload between team members						
	Maintenance of standards throughout procedure						
Decision-Making	Prompt diagnosis and assessment of problems						
	Implementation of and projection of decision						
Situational Awareness	Patient status perception						
	Anticipation and planning of required equipment throughout operation (e.g. basket, laser, stent)						
	Awareness of surrounding operating staff						
Resource Skills	Identification and resolution of potential stressors or distractors						
	Maintenance of other non-technical skills throughout stressors						

Feedback:

Score: N/A. Not Applicable 1. Unacceptable 2. Poor 3. Acceptable 4. Good 5. Excellent **Total Score:**
